# PCR for the detection of pathogens in neonatal early onset sepsis

**DOI:** 10.1371/journal.pone.0226817

**Published:** 2020-01-24

**Authors:** Clarissa Oeser, Marcus Pond, Philip Butcher, Alison Bedford Russell, Philipp Henneke, Ken Laing, Timothy Planche, Paul T. Heath, Kathryn Harris

**Affiliations:** 1 Paediatric Infectious Diseases, Institute of Infection and Immunity, St George’s, University of London, London, United Kingdom; 2 Molecular Microbiology, Institute of Infection and Immunity, St George’s, University of London, London, United Kingdom; 3 Neonatology, Birmingham Women's NHS Foundation Trust, Birmingham, United Kingdom; 4 Pediatric Infectious Disease and Rheumatology, University Medical Center Freiburg, Freiburg, Germany; 5 Microbiology, Virology and Infection Control, Great Ormond Street Hospital NHS Foundation Trust, London, United Kingdom; Universita degli Studi di Parma, ITALY

## Abstract

**Background:**

A large proportion of neonates are treated for presumed bacterial sepsis with broad spectrum antibiotics even though their blood cultures subsequently show no growth. This study aimed to investigate PCR-based methods to identify pathogens not detected by conventional culture.

**Methods:**

Whole blood samples of 208 neonates with suspected early onset sepsis were tested using a panel of multiplexed bacterial PCRs targeting *Streptococcus pneumoniae*, *Streptococcus agalactiae* (GBS), *Staphylococcus aureus*, *Streptococcus pyogenes* (GAS), *Enterobacteriaceae*, *Enterococcus faecalis*, *Enterococcus faecium*, *Ureaplasma parvum*, *Ureaplasma urealyticum*, *Mycoplasma hominis* and *Mycoplasma genitalium*, a 16S rRNA gene broad-range PCR and a multiplexed PCR for *Candida* spp.

**Results:**

Two-hundred and eight samples were processed. In five of those samples, organisms were detected by conventional culture; all of those were also identified by PCR. PCR detected bacteria in 91 (45%) of the 203 samples that did not show bacterial growth in culture. *S*. *aureus*, *Enterobacteriaceae* and *S*. *pneumoniae* were the most frequently detected pathogens. A higher bacterial load detected by PCR was correlated positively with the number of clinical signs at presentation.

**Conclusion:**

Real-time PCR has the potential to be a valuable additional tool for the diagnosis of neonatal sepsis.

## Introduction

Treatment for presumed sepsis is common in the neonatal period. Early onset sepsis (EOS) is defined as infection occurring within the first 48–72 hours of life, most often acquired shortly before or during delivery. The most common causal pathogen in the UK is *Streptococcus agalactiae* (GBS), followed by *Escherichia coli* [1].

Blood cultures remain the gold standard for diagnosing neonatal sepsis, in spite of being positive in a minority of cases with suspected sepsis, especially EOS [2, 3]. False negative blood cultures may occur as a result of small volumes of blood obtained, low levels of bacteraemia, use of prior (intrapartum) antibiotics or fastidious bacteria, fungi or viruses. Because a negative blood culture cannot exclude infection, antibiotic therapy is often continued beyond 48 hours despite negative blood cultures, especially, but not always, when there are clinical signs and laboratory markers consistent with infection [4]. This increases antibiotic usage, which in turn increases the risk of development of antibiotic resistant pathogens [5–8], as well as potential immune dysregulation in childhood, as a result of disruptions in nascent gut microbiome [9]. Moreover, prolonged empirical antibiotic therapy (>5 days) among neonates <1000g birth weight has been associated with an increased risk of death and necrotizing enterocolitis [10].

In an era of increasing antibiotic stewardship, improved diagnostic reliability is essential for limiting antibiotic usage. PCR-based techniques have the potential to improve diagnostic reliability.

In this study, whole blood samples of neonates with suspected EOS on the basis of risk and /or clinical indicators were processed by PCR methods including multiplexed, real-time PCR assays targeting a panel of bacterial pathogens, broad range bacterial 16S rRNA gene PCR and a multiplexed real-time PCR assay targeting *Candida* spp, in order to identify pathogens not detected by routine culture methods.

## Materials and methods

### Subjects

Neonates less than 72 hours of age undergoing investigation for presumed sepsis were recruited in three tertiary centres, following written parental consent. All neonates were inborn, evaluated either in the neonatal or maternity ward. The first-choice antibiotic regimen for empirical treatment of suspected EOS recommended by the National Institute for Health a and Care Excellence is bencylpenicillin with gentamicin. Investigations for sepsis could occur as a result of compatible clinical or laboratory signs or because of perceived risk factors in an otherwise asymptomatic neonate.

### Sample collection

Whole blood (0.5–1 ml) was collected in an EDTA bottle at the time of venipuncture when performing the initial sepsis screen or within 24 hours of initial evaluation. Confirmed infection was defined as a positive bacterial blood culture in the presence of clinical signs and symptoms of infection.

## Culture methods

Whole blood was collected using aseptic technique and cultured in BacT Alert Pediatric culture bottles for five days. As this was part of routine medical care of the patients and not the study procedure, blood volume collected was not recorded.

### DNA extraction

Total DNA was extracted from 100μl of whole blood using the Roche MagNA PURE automated extraction instrument (Roche Diagnostics, West Sussex, UK). DNA extracts were stored at -70°C.

### Bacterial multiplex PCR

Targets were bacteria known to account for the majority of EOS in developed countries [1, 11]. All extracts were processed by a panel of six bacterial multiplexed real-time PCR assays targeting *S*. *pneumoniae*, *S*. *agalactiae (GBS)*, *S*. *aureus*, *S*. *pyogenes* (GAS) / *Streptococcus pyogenes*, *Enterobacteriaceae*, *Enterococcus faecalis*, *Enterococcus faecium*, *Ureaplasma parvum*, *Ureaplasma urealyticum*, *Mycoplasma hominis* and *Mycoplasma genitalium* (see Table 1). The individual pathogen specific PCRs had been developed and evaluated previously [12–20]. For the purpose of this study, the efficiency of the reaction when a primer is combined with others was compared to the efficiency of the primer functioning on its own. Serial ten-fold dilutions of a known concentration of target DNA were amplified as monoplex and to test for inhibition, the resulting Ct values were compared to those required for detection of the same amount of target DNA in a triplex PCR. The reaction contained 13 μl of QuantiFast Multiplex mastermix (Qiagen, Crawley, United Kingdom) and 7μl of DNA extract. Primer and probes used for each reaction and their concentrations are shown in Table 1.

**Table 1:** 

Organism	Oligo Name	Sequence 5’ to 3’	Target gene	Conc (μM)	Reference
**Bacterial PCR**					
*Streptococcus pneumoniae*	LytA-F	ACG CAA TCT AGC AGA TGA AGC	LytA	0.2	Harris 2008 [12]
	LytA-R	TGT TTG GTT GGT TAT TCG TGC		0.2	
	LytA-Probe	FAM-TTT GCC GAA AAC GCT TGA TAC AGG G- BHQ1		0.2	
*Streptococcus agalactiae (GBS)*	GBS-F	ATC CTG AGA CAA CAC TGA CA	Sip	0.2	Berseng 2007 [13]
	GBS-R	TTG CTG GTG TTT CTA TTT TCA		0.2	
	GBS-Probe	JOE-ATC AGA AGA GTC ATA CTG CYA CTT C-BHQ1		0.2	Tann 2014 [14]
*Staphylococcus aureus*	SA _Forward	GTA GAT TGG GCA ATT ACA TTT TGG AGG	Coa	0.15	Sabet 2006 [15]
	SA _Reverse	CGC ATC TGC TTT GTT ATC CCA TGT A		0.15	
	SA _Probe	FAM- TAG GCG CAT TAG CAG TTG CAT C-BHQ1		0.15	
*Streptococcus pyogenes*	GAS_Forward	TGG ATG TGG TTG CAG GTT TAG AC	csrR	0.3	Tann 2014 [14]
	GAS_ Reverse	CGG GCA AGT AGT TCT TCA ATG G		0.3	
	GAS_Probe	JOE-CGG TGC AGA CGA CTA TAT TGT TAA ACC-BHQ1		0.2	
*Enterobacteriaceae* family	Ent_ Forward	ACCTGGGTACWACCAACTCTTGTGT	dnaK	0.3	Tann 2014 [14]
	Ent_Reverse	GTCACTGCCTGACGTTTAGC		0.3	
	Ent_Probe	JOE-AGGATGGTGAAACTCTGGTWGGTCAGCC-BHQ1		0.3	
*Enterococcus faecium*	Fium_Forward	TTC TTT GCT TTA TCC GAT GT	ddlfm	0.2	Mohn 2004 [16]
	Fium_Reverse	CGG TTT TCT GCT TTT GTA AT		0.2	
	Fium_Probe	FAM- ACT AGA ACC CAT ATT CGC C-BHQ1		0.15	
*Enterococcus faecalis*	Falis_Forward	CGCTTCTTTCCTCCCGAGT	16S	0.24	Santo Domingo 2003 [17]
	Falis_Reverse	GCCATGCGGCATAAACTG		0.24	
	Falis_Probe	Hex-GAGGAGTGGCGGACG-BHQ1		0.15	
*Ureaplasma parvum*	UPure F	CAT TGA TGT TGC ACA AGG AGA AA	ure	0.24	Cao 2009 [18]
	UPure R	TTA GCA CCA ACA TAA GGA GCT AAA TC		0.24	
	UPure Probe	FAM-TTG ACC ACC CTT ACG AG-BHQ1		0.15	
*Ureaplasma urealyticum*	UUre_F	ATC GAC GTT GCC CAA GGG GA	ure	0.24	Cao 2009 [18]
	UUre R	TTA GCA CCA ACA TAA GGA GCT AAA TC		0.24	
	UUre Probe	HEX-TTG TCC GCC TTT ACG AG–BHQ1		0.15	
*Mycoplasma genitalium*	MgPa-355F	GAGAAATACCTTGATGGTCAGCAA	G-37T	0.25	Jensen 2004 [19]
	MgPa-432R	GTTAATATCATATAAAGCTCTACCGTTGTTATC		0.25	
	MgPa-380	FAM-ACTTTGCAATCAGAAGGT-MGB		0.15	
*Mycoplasma hominis*	MHyidCfwd	TCA CTA AAC CGG GTA TTT TCT AAC AA	yidC	0.25	Ferandon 2010 [20]
	MHyidCrev	TTG GCA TAT ATT GCG ATA GTG CTT		0.25	
	MHyidC	HEX- CTA CCA ATA ATT TTA ATA TCT GTC GGT ATG-BHQ1		0.15	
Internal positive control (added in A, B, C, D, E and F)	IPC _F	GGA CAC TAT GCC CCT CCT TAG A	mus	0.1	Tann 2014 [14]
	IPC _R	AGC TCC AAA CTC CGT CTC TGT AA		0.1	
	IPC _Probe	CY5-TTG GGA ACA AAA CAC CCA TGG AAG GA-BHQ3		0.1	
***Candida* PCR**					
*Candida* spp.	cand-CR1 (forward)	CGGGTGGGAAATTCGGT	RPR1	0.1	Innings 2007 [22]
	cand-CR5 (reverse)	CAATGATCGGTATCGGGT		0.1	
	cand-ROX (reverse)	ROX- TTCGCATATTgCAcTAAaYaGa–BHQ2*		0.1	
*C*. *glabrata*	gla-CR3 (forward)	RGCAACGGCTGGGAAT		0.1	
	cand-CR5 (reverse)	CAATGATCGGTATCGGGT		0.1	
	gla-JOE (reverse)	JOE-TAAAGCCTCACCACGATTTTGACAC- BHQ1		0.1	
*C*. *krusei*	cand-CR1 (forward)	CGGGTGGGAAATTCGGT		0.1	
	krus-CR5 (reverse)	TAGTGATCGGTATCGAGTT		0.1	
	krus-Cy5 (reverse)	Cy5- CCAAAGTTGTACAAGCAAGTACCA- BHQ3		0.1	
*C*. *albicans*	cand-CR1 (forward)	CGGGTGGGAAATTCGGT		0.1	
	cand-CR5 (reverse)	CAATGATCGGTATCGGGT		0.1	
	alb-FAM (reverse)	Fam- CAGCTTGTAGTAAAGAATTACTCAC-BHQ1		0.1	

Primers and probes as used in the PCR panel. Each PCR was run as a triplex PCR with an internal positive control. The table lists their sequence, including the probes’ fluorophores (FAM, JOE, CY5) and quenchers (BHQ1 and BHQ2), the target gene, melt temperature (Tm) and guanine cytosine content (GC).

A positive internal control was added to every sample prior to extraction to control for extraction efficiency and PCR inhibition. Every batch of samples tested included a negative and positive control. Thermocycling was performed on the BioRad CFX 96 Real Time PCR detection system (BioRad, Hertfordshire, UK) as follows: 5 minutes at 95°C followed by 45 cycles of 15 seconds at 95°C and 30 seconds at 60°C.

A positive PCR signal was defined as quantification cycle (Cq) ≤40. Amplicons of samples testing positive for *Enterobactericeae* were sequenced. Only samples yielding sequences that could be identified to at least genus level using BLAST analysis against the Genbank database were considered true positives.

### 16S rRNA gene PCR

Samples testing negative by bacterial multiplex PCR were processed by 16S rRNA gene PCR using Power SYBR® Green PCR Master Mix (Life Technologies, Paisley, UK). 5μl extracted DNA was added to 12.5 μl Master mix, 0.1 μM of forward primer 16SFa (GCTCAGATTGAACGCTGG), 0.05 μM each of forward primer 16SFb1 and 16SFb2 (GCTCAGGACGAACGCTGG and GCTCAGGATGAACGCTGG), 0.1 μM of reverse primer 16SR (ACTGCTGCCTCCCGTA) [21] and 6.5μl molecular grade nuclease free water (Qiagen). This primer pair covers the highly variable region V1-V2, amplifying a PCR product of approximately 320 base pairs. This primer pair had been developed for routine use in Great Ormond Street Children Hospital clinical microbiology laboratory for diagnosis of culture negative samples and identification of unknown isolates [21].

### *Candida* multiplex PCR

Samples from which no bacterial pathogen was identified were further processed by a multiplexed real-time PCR targeting *C*. *albicans*, *C*. *glabrata*, *C*. *krusei*, *C*. *dubliniensis*, *C*. *famata*, *C*. *guilliermondii*, *C*. *parapsilosis and C*. *tropicalis* [22]. The reaction contained 13 μl of QuantiFast Multiplex mastermix (Qiagen), 7μl of extracted DNA, and 0.1 each of primers (cand-CR1; cand–CR5) and 0.1 uM probe (cand-rox) (Table 1).

### Sequencing

For positive *Enterobacteriaceae* and 16S rRNA gene PCR assays the resulting amplicon was sequenced using Big-dye 3.1 sequencing kit (Thermo Fisher, Paisley, UK) and run on a 3130 Genetic analyser (ThermoFisher).

### Clinical observations

Clinical signs to be recorded at enrolment were specified in the study proforma and included fever, hypothermia, respiratory distress, grunting, nasal flare, tachypnoea, recession/retraction, hypoxia, tachycardia, bradycardia, poor perfusion, hypotension, poor feeding, irritability, hypotonia, convulsions, apnoea, lethargy, metabolic acidosis, glucose imbalance, high white cell count (WCC), neutrophil count and raised C-reactive protein (CRP)

Fever was defined as axillary / central temperature of ≥38°C on one occasion or ≥37.5°C on two occasions separated by at least one hour, A raised CRP was defined as > 10mg/L, a raised WCC as (> 20 000 x10^9^ cells/L), glucose imbalance as ≤2.2 /≥10mmol/l for 4 hours in spite of corrective measures and metabolic acidosis as base excess (BE) ≥-8 mmol/L over 4 hours in spite of corrective measures.

To correlate the number of clinical signs and laboratory markers with bacterial load samples detected by qPCR, samples were grouped by their PCR signal into strongly positive (< 35 Cq), positive (35–38 Cq), weakly positive (38–40 Cq) and negative (>40 Cq or no signal) as is routine in clinical practice. In samples positive for multiple bacteria only the strongest signal was taken into account. The groups were compared using a two-tailed Fisher’s exact test (GraphPad Software, La Jolla California, USA).

## Results

### Clinical observations

Blood samples from 208 neonates were analyzed. Fifty-nine (28%) were preterm (< 37 weeks gestational age), with 12 (6%) very preterm (28 to < 32 weeks) and 11 (5%) extremely preterm (< 28 weeks). Sixty mothers had received intrapartum antibiotics. One or more clinical signs were observed in 147 (71%) of all neonates: 17 (6 signs), 15 (5 signs), 19 (4 signs), 30 (3 signs), 36 (2 signs) and 30 (1 sign). The most commonly occurring signs were respiratory: tachypnoea, respiratory distress, grunting or recession. Sixty-five (31%) neonates had an elevated CRP. Of those, 27 neonates had an elevated CRP on initial screen and also on the following day, whilst 26 neonates had an elevated CRP only on the second day. A repeat CRP was not performed in all neonates; in 107 (51%) CRP results were only available from the day of the initial screen. An elevated white cell count was reported in 46 neonates (22%). All neonates survived to hospital discharge.

### Culture positivity

Samples from 5 neonates were culture positive and all were also positive by PCR for the same organism (four GBS and one *E*. *faecalis)*. The remaining 203 samples were culture negative after five days of incubation and PCR detected an organism in 91 (45%) of these. The five infants ranged in gestation from 39 to 42 weeks. One baby had six clinical signs, two five and the remaining two had four clinical signs, ranging from fever, grunting, tachypnoea, recession, hypoxia, irritability, hypotonia and convulsions to glucose imbalance. In one of the five babies, the mother had received antenatal antibiotics.

### Bacterial PCR

Samples from 208 neonates were tested by the bacterial real-time PCR panel and samples from 96 neonates tested positive for at least one bacterial species. Of those neonates with a positive bacterial PCR, 22 of 96 (23%) were born prematurely and 25 of their mothers (26%) had received intrapartum antibiotics. Clinical signs and/or laboratory results indicative of sepsis were present in 80 (83%); 69 (72%) had clinical signs; 34 (35%) had an elevated CRP and 23 (24%) had an abnormal white cell count. 18 samples tested positive for more than one bacterium. Seven of the 29 with no recorded clinical signs had an elevated CRP (one of them also had an elevated WCC), a further six had an elevated WCC.

Of those neonates with a negative culture and a negative bacterial PCR (n = 112), 87 (77%) had clinical signs consistent with sepsis. Of the 32 with no clinical signs, seven had an elevated CRP and/or WCC.

Newborns with a positive PCR result did not differ significantly from those with a negative PCR result in term of clinical signs, laboratory markers prematurity or maternal antibiotic with the exception of hypothermia, which was significantly more common amongst infants with a positive PCR result (see Table 2).

**Table 2:** 

	PCR +ve % (N)	PCR -ve % (N)	p-value
Prematurity	23 (22)	32 (36)	0.25
Maternal antibiotics	26 (25)	33 (37)	0.47
Total symptomatic	83 (80)	77 (87)	0.48
Tachypnoea	43 (41)	40 (44)	0.67
Hypothermia	31 (30)	2 (2)	0.00026
Recession/retraction	25 (24)	28 (31)	0.74
Grunting	22 (21)	29 (33)	0.94
Fever	17 (16)	17 (19)	0.97
Nasal flare	11 (11)	13 (14)	0.90

Most common signs and clinical features of newborns testing PCR positive vs negative.

**Fig 1:**
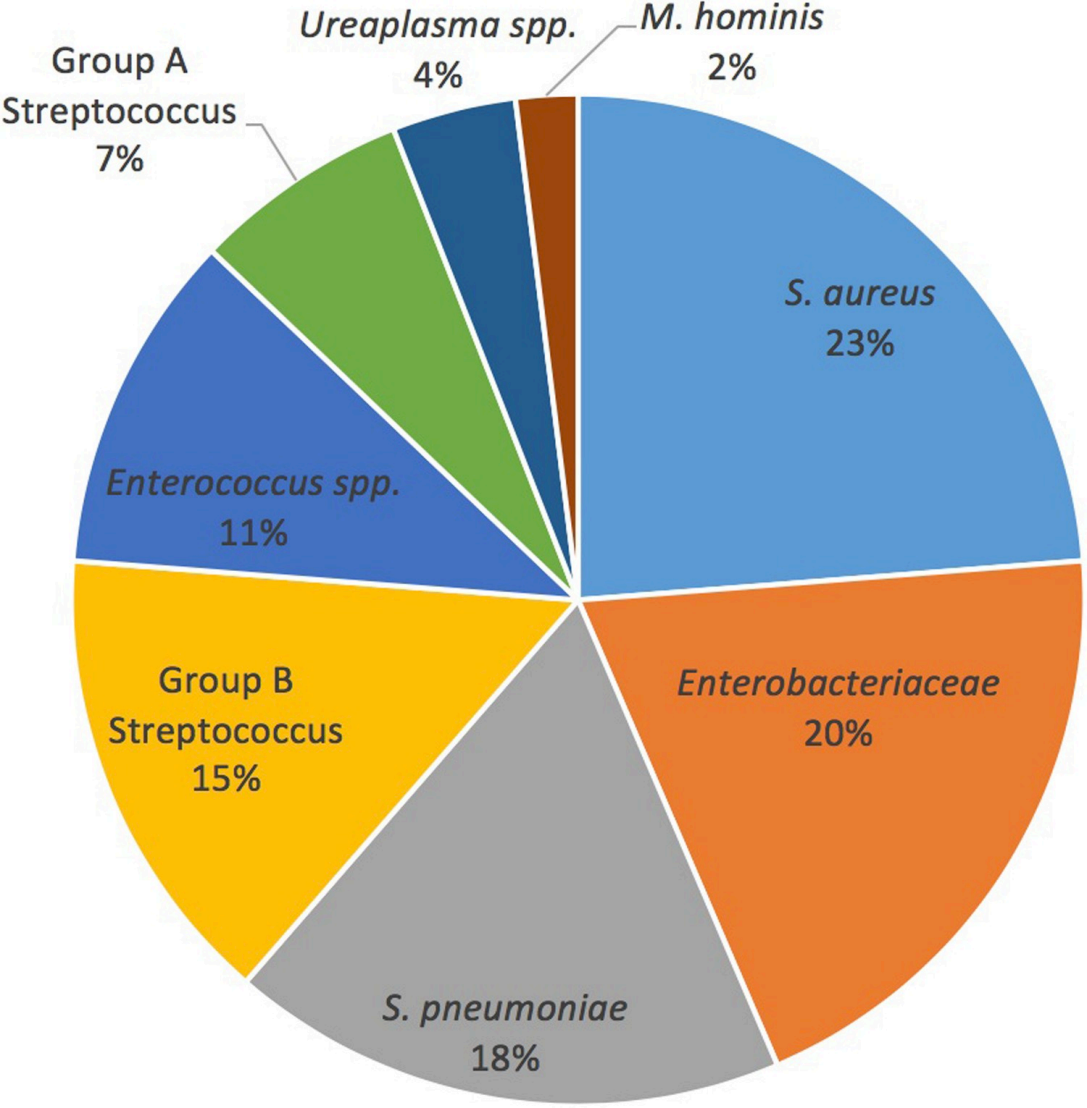
Distribution of bacteria detected by specific bacterial PCR in 96 samples of neonates with suspected EOS.

The distribution of bacterial species detected by multiplex PCR is described in Fig 1 and Table 3. The most frequently detected organisms were *S*. *aureus* in 28 samples (24%), *Enterobacteriaceae* in 23 samples (20%), *S*. *pneumoniae* in 21 samples (18%) and GBS in 17 samples (15%).

**Table 3:** 

Pathogen	<35 Cq (strongly positive)	35–38 Cq (positive)	38–40 Cq (weakly positive)	% (N)
*S*. *aureus*	4	17	7	24 (28)
*Enterobacteriaceae*	5	18	0	20 (23)
*S*. *pneumoniae*	1	16	4	18 (21)
*Streptococcus agalactiae*	10	5	2	15 (17)
*Enterococcus* spp.^1^	4	7	2	11 (13)
GAS	2	4	2	7 (8)
*Ureaplasma* spp. ^2^	1	3	1	4 (5)
*M*. *hominis*	1	1	0	2 (2)
**Total [% (n)]**	**24 (28)**	**60 (71)**	**15 (18)**	**100 (117)**

Distribution of bacteria detected by multiplex PCR by Cq range. The total number of bacteria (117) is higher than the number of samples positive for multiplex PCR (96) as 18 samples contained multiple bacteria.

^1^ 10 *E*. *faecalis*, 3 *E*. *faecium*.

^2^ 4 *U*. *parvum*, 1 *U*. *urealyticum*.

#### Correlation between clinical signs / laboratory markers and PCR results

Table 4 describes the association between clinical and laboratory features of infants with presumed sepsis and their PCR results. Neonates with strongly positive signals (<35 Cq) were likely to have more clinical signs than those with positive, weak or no signals. This was particularly so for strongly positive vs. positive signals for those with ≥ 1 sign (p = 0.005) and ≥ 5 signs (p = 0.009). Associations between abnormal CRP and WCC values with PCR results were less apparent.

**Table 4:** 

	Cq <35 % (N)	<35 vs 35–38 p-value	Cq = 35–38 % (N)	35–38 vs 38–40 p-value	Cq = 38–40 % (N)	38–40 vs >40 p-value
Laboratory markers						
CRP	50 (12)	0.08	29 (17)	0.74	36 (5)	0.34
WCC	25 (6)	1.00	27 (16)	0.16	7 (1)	0.30
Clinical signs						
≥1 sign	88 (21)	0.005	54 (32)	0.37	71 (10)	1
≥2 signs	75 (18)	0.02	46 (27)	1.00	43 (6)	0.27
≥3 signs	60 (14)	0.05	32(19)	1.00	29 (4)	0.56
≥ 4 signs	42 (10)	0.19	25 (15)	1.00	21 (3)	1.00
≥ 5 signs	38 (9)	0.009	10 (6)	0.64	14 (2)	1.00
≥ 6 signs	13 (3)	0.35	5 (3)	0.24	14 (2)	0.35

Correlation between clinical signs / laboratory markers and PCR results. This table shows the percentage (number) of infants with samples in each Cq range and number of clinical signs (1 to 6) as well as percentage (number) of infants with abnormal laboratory markers and the p-values as calculated by two-tailed Fisher’s exact test.

Twenty-seven samples tested positive for *Enterobacteriaceae* by PCR and the resulting amplicons were sequenced. In 23 of these a single organism was identified by BLAST analysis to at least genus level whilst in the remaining four, sequence data appeared mixed or no data was produced. *E*. *coli* and *Enterobacter* spp. were detected most frequently (see Table 5).

**Table 5:** 

Amplicon sequencing results for pan-*Enterobacteriacae* PCR positive samples	N
*E*.*coli*	7
*Enterobacter* spp.	5
*Enterobacter* spp.*/Citrobacter* spp.	3
*Klebsiella* spp.	2
*Klebsiella* spp.*/E*.*coli*	1
*Klebsiella* spp.*/ Enterobacter*	1
*Citrobacter* spp.	1
*Citrobacter* spp.*/Pantoea* spp.	2
*Serratia* spp.	1
**Total**	**23**

Enterobacteriacae identified by Sanger sequencing of the Enterobacteriaceae dnaK PCR product from 23 samples.

In 18/117 (15%) of positive samples more than one pathogen was detected; 15 samples tested positive for 2 bacterial species and 1 sample each tested positive for 3 and 4 bacterial species. Table 6 shows the combinations of bacteria found together.

**Table 6:** 

Polymicrobial infection	No
*S*. *pneumoniae* + *S*. *aureus*	5
*S*. *pneumoniae* + GBS	2
*S*. *aureus* + *Enterobacteriaceae*	2
*S*. *aureus* + *Enterococcus* spp.	2
*S*.*pneumoniae* + *S*. *pyogenes*	1
*S*.*pneumoniae* + *Enterobacteriaceae*	1
GBS + *Enterobacteriaceae*	1
*U*. *parvum* + *S*. *aureus*	1
GBS *+* GAS	1
*S*. *pneumoniae* + *S*. *agalactiae* + *E*. *faecalis*	1
*S*. *pneumoniae* + *S*. *aureus* + *Enterococcus* spp. + *S*. *agalactiae*	1
Total	18

Combinations of bacteria found in 18 samples positive for more than one organism and their numbers.

### 16S rRNA gene PCR

In total 108 samples were processed by 16S rRNA gene PCR; these included samples positive for *Enterobacteriacae* for which sequence-based identification could not be achieved.

Of these, 13 were 16S rRNA gene PCR positive and sequencing of the PCR product identified *Streptococcus* spp. (mitis group) in two cases and *Propionibacterium* spp. in three cases. In four cases, waterborne organisms *(Pseudomonas* spp./ *P*. *fluorescens*), likely to be contaminants, were identified; the remaining four cases showed a mixed or no sequence data.

### *Candida* multiplex PCR

Of the 60 samples that were negative by every other test, 36 had sufficient residual extract for testing with the Candida multiplexed PCR. All tested negative.

## Discussion

A large number of neonates are treated with antibiotics for presumed infection despite having no organism detected in their blood cultures.

A recent metanalysis on molecular assays for the diagnosis of sepsis in neonates reports a mean sensitivity of 0.90 and specificity of 0.93. The authors conclude that molecular assays are feasible in neonates and due to their rapid detection times compared to blood cultures may impact on early diagnosis and treatment [23].

This study investigated blood samples of neonates with suspected EOS and demonstrated that a panel of multiplexed real-time PCR assays could identify pathogens in 45% of cases where blood cultures were negative as well as correctly identifying a pathogen in 100% of cases where blood cultures were positive. Furthermore, we have shown that higher bacterial load appears to correlate with the number of clinical signs at presentation. No significant association was detected between an abnormal CRP and PCR results, however this study was conducted before current NICE guidelines were implemented, which recommend to perform a repeat CRP at 18–24 hours [24]. A CRP value from a sample obtained too early in life could represent a false negative. Only half of all neonates had results of a second CRP available, therefore a complete evaluation of this association was not possible.

The samples of this study were subject to some limitations. Extraction was performed on a robotic platform, a technique shown to be inferior to manual extraction in terms of DNA yield and purity [25]. Extracts were stored at -70° Celsius until final processing. Storage conditions can have significant impact on DNA integrity [26] and extracts are especially vulnerable to repeated freeze/thaw cycles [27]. A number of samples were subjected to repetitions of the different processing steps; potentially increased contamination and/or degradation could have occurred each time.

Some members of the *Enterobacteriaceae* family are ubiquitous skin and bowel commensals and environmental contaminants. The high number of these bacteria detected raises suspicion of contamination and amongst samples positive for *Enterobacteriaceae* only those yielding a sequence identifiable to genus level by BLAST analysis were therefore reported. This was based on the previous observation that contamination usually occurs with multiple species from this family (K. Harris–unpublished data).

Overall, *S*. *aureus* was the most frequently detected organism (23%) and even though generally considered as representing true infection [28], early infant colonization is not uncommon [29] and thus skin contamination may also have contributed to the high number found in this study.

Nonetheless, with cautious interpretation of these data, this study has notable findings. Most significantly, a large proportion of samples (45%) tested positive by bacterial multiplex PCR. In contrast, a pathogen was identified by routine culture methods in only 2.4%.

*S*. *pneumoniae* was present in 18% of all positive samples, more frequent than GBS (15%). *S*. *pneumoniae* is a relatively rare pathogen in neonatal sepsis (1–11%) [30], and a rare cause of neonatal skin colonization, yet when it occurs, the course of the disease has been reported to be more severe, particularly in EOS [31]. Early onset *S*. *pneumoniae* infection is often associated with maternal vaginal colonization or disease [32–34]. The bacterium can be difficult to culture, particularly following administration of intrapartum antibiotics [12, 35], which could lead to under diagnosis based on blood culture data. Thus this study might reflect a more accurate representation of its prevalence in newborn sepsis.

*Enterococcus* spp. were detected in 11% of all PCR positive cases. Of concern is that only in one case was it isolated by blood culture, despite there being no general awareness of difficulties in culturing these bacteria. An increase of *Enterococcus* spp. in neonatal units has been reported [36] and concerns regarding the spread of vancomycin-resistant strains has prompted many units to adopt surveillance and control measures [37, 38].

GAS is considered a neonatal pathogen of the past [39]. However, PCR methods detected GAS in eight samples (7%). Similarly, *Ureaplasma* spp. and *Mycoplasma* spp., which have been associated with premature birth and infection [40], are rarely detected in cultures due to their fastidious growth requirements. PCR was able to identify these organisms in seven cases. Increased detection of these pathogens, together with further evidence of their significance, could have implications for treatment regimens.

A large number of samples (15%) tested positive for multiple bacteria. The proportion of mixed infections reported by culture based studies range from 1–19%. [1, 41–45]. These results, to some extent, may represent contamination, as discussed above. However, polymicrobial bloodstream infections are important, as they are associated with a more than 3-fold increase in mortality, increase in duration of infection and greater severity of illness [45,46].

The correlation between the number of clinical signs and PCR positivity is a novel finding and provides some confidence in the relevance of these tests. Clinical signs and laboratory markers are known to be subjective and non-specific in the context of neonatal sepsis [47] but have not been validated using PCR as gold standard. The only individual sign to show significance between PCR positive and negative cases was hypothermia, strengthening its value as a marker of sepsis. Clinical signs of sepsis to evaluate sepsis in this study are therefore also nonspecific and included signs of respiratory distress which may reflect different pathologies particularly in the premature infant. Our data suggests that use of PCR could provide insight into the value of different (and the number of) clinical signs and biomarkers in diagnosing neonatal sepsis.

## Conclusions

Molecular diagnostic methods are capable of detecting a large number of pathogens in samples from neonates with suspected sepsis. False positive results can have significant implications for clinical practice as well as research. Therefore strict procedures for sample collection and processing to avoid contamination need to be applied, as well as cautious interpretation of results particularly when organisms associated with skin or environmental contamination are detected. In this study, in contrast to many studies based on culture, GBS was not the most commonly detected pathogen in early onset sepsis. Indeed, *S*. *pneumoniae* may be a more important pathogen in EOS than the existing literature indicates. Polymicrobial sepsis might also occur more often than currently estimated. As an additional diagnostic tool, PCR methods have the potential to increase diagnostic reliability of causal pathogens for neonatal sepsis. This could facilitate a reduction in unnecessary broad spectrum antibiotic usage, and target treatment to improve outcomes, as well as limit the development of antibiotic resistance. Future studies could also explore associations of clinical signs and PCR positivity from different organisms.

## References

[1] Vergnano S, Menson E, Kennea N, Embleton N, Russell AB, Watts T, et al Neonatal infections in England: the NeonIN surveillance network. Arch Dis Child Fetal Neonatal Ed. 2011; 96(1): p. F9–F14. 10.1136/adc.2009.178798 20876594

[2] Stocker M, Fontana M, El Helou S, Wegscheider K, Berger TM. Use of procalcitonin-guided decision-making to shorten antibiotic therapy in suspected neonatal early-onset sepsis: prospective randomized intervention trial. Neonatology 2010; 97(2): p. 165–74. 10.1159/000241296 19776651

[3] Blackburn RM, Muller-Pebody B, Planche T, Johnson A, Hopkins S, Sharland M, et al Neonatal sepsis—many blood samples, few positive cultures: implications for improving antibiotic prescribing. Arch Dis Child Fetal Neonatal Ed. 2012; 97(6): p. F487–8. 10.1136/archdischild-2012-302261 22762988

[4] Wirtschafter DD, Padilla G, Suh O, Wan K, Trupp D, Fayard EE. Antibiotic use for presumed neonatally acquired infections far exceeds that for central line-associated blood stream infections: an exploratory critique. J Perinatol. 2011; 31(8): p. 514–8. 10.1038/jp.2011.39 21546938

[5] Patel SJ, Saiman L. Principles and strategies of antimicrobial stewardship in the neonatal intensive care unit. Semin Perinatol. 2012; 36(6): p. 431–6. 10.1053/j.semperi.2012.06.005 23177802 PMC3509381

[6] Patel SJ, Saiman L. Antibiotic resistance in neonatal intensive care unit pathogens: mechanisms, clinical impact, and prevention including antibiotic stewardship. Clin Perinatol. 2010; 37(3): p. 547–63. 10.1016/j.clp.2010.06.004 20813270 PMC4440667

[7] Macharashvili N, Kourbatova E, Butsashvili M, Tsertsvadze T, McNutt LA, Leonard MK. Etiology of neonatal blood stream infections in Tbilisi, Republic of Georgia. Int J Infect Dis. 2009; 13(4): p. 499–505. 10.1016/j.ijid.2008.08.020 19058989 PMC2695829

[8] Yilmaz NO, Agus N, Helvaci M, Kose S, Ozer E, Sahbudak Z. Change in Pathogens Causing Late-onset Sepsis in Neonatal Intensive Care Unit in Izmir, Turkey. Iran J Pediatr. 2010; 20(4): p. 451–8. 23056745 PMC3446087

[9] Meropol SB, Edwards A. Development of the infant intestinal microbiome: A bird's eye view of a complex process. Birth Defects Research Part C Embryo Today Reviews 2015 105(4) 10.1002/bdrc.21114 PMC5637388 26663826

[10] Cotten CM, Taylor S, Stoll B, Goldberg RN, Hansen NI, Sánchez PJ, et al Prolonged duration of initial empirical antibiotic treatment is associated with increased rates of necrotizing enterocolitis and death for extremely low birth weight infants. Pediatrics 2009, 123(1): p. 58–66. 10.1542/peds.2007-3423 19117861 PMC2760222

[11] Muller-Pebody B, Johnson AP, Heath PT, Gilbert RE, Henderson KL, Sharland M; iCAP Group. Empirical treatment of neonatal sepsis: are the current guidelines adequate? Arch Dis Child Fetal Neonatal Ed. 2011; 96(1): p. F4–8. 10.1136/adc.2009.178483 20584804

[12] Harris KA, Turner P, Green EA, Hartley JC. Duplex real-time PCR assay for detection of Streptococcus pneumoniae in clinical samples and determination of penicillin susceptibility. J Clin Microbiol. 2008; 46(8): p. 2751–8. 10.1128/JCM.02462-07 18562586 PMC2519471

[13] Berseng H, Bevanger L, Rygg M, Bergh K. Real-time PCR targeting the sip gene for detection of group B Streptococcus colonization in pregnant women at delivery. J Med Microbiol. 2007 2;56(Pt 2):223–8. 10.1099/jmm.0.46731-0 17244804

[14] Tann C, Nkurunziza P, Nakakeeto M, Oweka J, Kurinczuk JJ, Were J et al Prevalence of Bloodstream Pathogens Is Higher in Neonatal Encephalopathy Cases vs. Controls Using a Novel Panel of Real-Time PCR Assays. PLoS One. 2014; 9(5): e97259 10.1371/journal.pone.0097259 24836781 PMC4023955

[15] Sabet NS, Subramaniam G, Navaratnam P, Sekaran SD. Simultaneous species identification and detection of methicillin resistance in staphylococci using triplex real-time PCR assay. Diagn Microbiol Infect Dis. 2006 9;56(1):13–8 10.1016/j.diagmicrobio.2006.02.013 16650954

[16] Mohn SC, Ulvik A, Jureen R, Willems RJ, Top J, Leavis H et al Duplex real-time PCR assay for rapid detection of ampicillin-resistant Enterococcus faecium. Antimicrob Agents Chemother. 2004 2;48(2):556–60. 10.1128/AAC.48.2.556-560.2004 14742209 PMC321536

[17] Santo Domingo JW, Siefring SC, Haugland RA. Real-time PCR method to detect Enterococcus faecalis in water. Biotechnol Lett 2003 2;25(3):261–5. 10.1023/a:1022303118122 12882582

[18] Cao X, et al, Real-time TaqMan polymerase chain reaction assays for quantitative detection and differentiation of Ureaplasma urealyticum and Ureaplasma parvum. Diagn Microbiol Infect Dis, 2007 57(4): p. 373–8. 10.1016/j.diagmicrobio.2006.09.006 17141453

[19] Jensen JS, Björnelius E, Dohn B, Lidbrink P. Use of TaqMan 5' nuclease real-time PCR for quantitative detection of Mycoplasma genitalium DNA in males with and without urethritis who were attendees at a sexually transmitted disease clinic. J Clin Microbiol, 2004 42(2): p. 683–92. 10.1128/JCM.42.2.683-692.2004 14766837 PMC344445

[20] Ferandon C, Peuchant O, Janis C, Benard A, Renaudin H, Pereyre S, et al, Development of a real-time PCR targeting the yidC gene for the detection of Mycoplasma hominis and comparison with quantitative culture. Clin Microbiol Infect, 2011 17(2): p. 155–9 10.1111/j.1469-0691.2010.03217.x 20298269

[21] Harris KA, Hartley JC. Development of broad-range 16S rDNA PCR for use in the routine diagnostic clinical microbiology service. J Med Microbiol. 2003; 52(Pt 8): p. 685–91. 10.1099/jmm.0.05213-0 12867563

[22] Innings A, Ullberg M, Johansson A, Rubin CJ, Noreus N, Isaksson M, et al Multiplex real-time PCR targeting the RNase P RNA gene for detection and identification of Candida species in blood. J Clin Microbiol. 2007; 45(3): p. 874–80. 10.1128/JCM.01556-06 17215340 PMC1829127

[23] Pammi M, Flores A, Versalovic J, Leeflang MMG. Molecular assays for the diagnosis of sepsis in neonates. Cochrane Database of Systematic Reviews 2017; 2 CD011926. 10.1002/14651858.CD011926.pub2 28236648 PMC6464551

[24] Simonsen KA, Anderson-Berry AL, Delair SF, Davies HD. Early-onset neonatal sepsis. Clin Microbiol Rev. 2014; 27(1): p. 21–47. 10.1128/CMR.00031-13 24396135 PMC3910904

[25] Riemann K, Adamizik M, Frauenrath S, Egensperger R, Schmid KW, Brockenmeyer NH, et al Comparison of manual and automated nucleic acid extraction from whole-blood samples. J Clin Lab Anal. 2007; 21(4): p. 244–8. 10.1002/jcla.20174 17621359 PMC6649159

[26] Roder B, Fruhwirt K, Vogl C, Wagner M, Rossmanith P. Impact of long-term storage on stability of standard DNA for nucleic acid-based methods. J Clin Microbiol. 2010; 48(11): p. 4260–2. 10.1128/JCM.01230-10 20810770 PMC3020885

[27] Ross KS, Haites NE, Kelly KF. Repeated freezing and thawing of peripheral blood and DNA in suspension: effects on DNA yield and integrity. J Med Genet. 1990; 27(9): p. 569–70. 10.1136/jmg.27.9.569 2231649 PMC1017219

[28] Hall KK, Lyman JA. Updated review of blood culture contamination. Clin Microbiol Rev. 2006; 19(4): p. 788–802. 10.1128/CMR.00062-05 17041144 PMC1592696

[29] Jimenez-Truque N, Tedeschi S, Saye EJ, McKenna BD, Langdon W, Wright JP, et al Relationship between maternal and neonatal Staphylococcus aureus colonization. Pediatrics 2012; 129(5): p. e1252–9. 10.1542/peds.2011-2308 22473373 PMC3340589

[30] Hoffman JA, Mason EO, Schutze GE, Tan TQ, Barson WJ, Givner LB, et al Streptococcus pneumoniae infections in the neonate. Pediatrics 2003; 112(5): p. 1095–102. 10.1542/peds.112.5.1095 14595052

[31] Gomez M, Alter S, Kumar ML, Murphy S, Rathore MH. Neonatal Streptococcus pneumoniae infection: case reports and review of the literature. Pediatr Infect Dis J. 1999 18(11): p. 1014–8. 10.1097/00006454-199911000-00016 10571441

[32] Simpson JM, Patel JS, Ispahani P. Streptococcus pneumoniae invasive disease in the neonatal period: an increasing problem? Eur J Pediatr.1995; 154(7): p. 563–6. 10.1007/bf02074835 7556324

[33] Rhodes PG, Burry VF, Hall RT, Cox R. Pneumococcal septicemia and meningitis in the neonate. J Pediatr.1975; 86(4): p. 593–5. 10.1016/s0022-3476(75)80159-4 236367

[34] Sallam A, Paes B. Streptococcus pneumoniae: an old bug with significant maternal-newborn implications. Am J Perinatol. 2004; 21(8): p. 491–5. 10.1055/s-2004-835967 15580546

[35] Petti CA, Woods CW, Reller LB. Streptococcus pneumoniae antigen test using positive blood culture bottles as an alternative method to diagnose pneumococcal bacteremia. J Clin Microbiol. 2005; 43(5): p. 2510–2. 10.1128/JCM.43.5.2510-2512.2005 15872298 PMC1153727

[36] McNeeley DF, Saint-Louis F, Noel GJ. Neonatal enterococcal bacteremia: an increasingly frequent event with potentially untreatable pathogens. Pediatr Infect Dis J. 1996; 15(9): p. 800–5. 10.1097/00006454-199609000-00013 8878225

[37] Sherer CR, Sprague BM, Campos JM, Nambiar S, Temple R, Short B, et al Characterizing vancomycin-resistant enterococci in neonatal intensive care. Emerg Infect Dis. 2005; 11(9): p. 1470–2. 10.3201/eid1109.050148 16229786 PMC3310622

[38] Singh N, Léger MM, Campbell J, Short B, Campos JM. Control of vancomycin-resistant enterococci in the neonatal intensive care unit. Infect Control Hosp Epidemiol. 2005; 26(7): p. 646–9. 10.1086/502595 16092746

[39] Greenberg D, Leibovitz E, Shinnwell ES, Yagupsky P, Dagan R. Neonatal sepsis caused by Streptococcus pyogenes: resurgence of an old etiology? Pediatr Infect Dis J.1999; 18(5): p. 479–81. 10.1097/00006454-199905000-00021 10353530

[40] Waites KB, Katz B, Schelonka RL. Mycoplasmas and ureaplasmas as neonatal pathogens. Clin Microbiol Rev. 2005; 18(4): p. 757–89. 10.1128/CMR.18.4.757-789.2005 16223956 PMC1265909

[41] Yapicioglu H, Ozcan K, Sertdemir Y, Mutlu B, Satar M, Narli N, et al Healthcare-associated infections in a neonatal intensive care unit in Turkey in 2008: incidence and risk factors, a prospective study. J Trop Pediatr. 2011; 57(3): p. 157–64. 10.1093/tropej/fmq060 20601690

[42] Ronnestad A, Abrahamsen TG, Medbø S, Reigstad H, Lossius K, Kaaresen PI, et al Septicemia in the first week of life in a Norwegian national cohort of extremely premature infants. Pediatrics 2005; 115(3): p. e262–8. 10.1542/peds.2004-1834 15687417

[43] Olsen AL, Reinholdt J, Jensen AM, Andersen LP, Jensen ET. Nosocomial infection in a Danish Neonatal Intensive Care Unit: a prospective study. Acta Paediatr. 2009; 98(8): p. 1294–9. 10.1111/j.1651-2227.2009.01322.x 19438843

[44] Wojkowska-Mach J, Borszewska-Kornacka M, Domańska J, Gadzinowski J, Gulczyńska E, Helwich E, et al Early-onset infections of very-low-birth-weight infants in Polish neonatal intensive care units. Pediatr Infect Dis J. 2012; 31(7): p. 691–5. 10.1097/INF.0b013e3182567b74 22466319

[45] Pammi M, Zhong D, Johnson Y, Revell P, Versalovic J. Polymicrobial bloodstream infections in the neonatal intensive care unit are associated with increased mortality: a case-control study. BMC Infect Dis. 2014; 14: p. 390 10.1186/1471-2334-14-390 25022748 PMC4226990

[46] Tsai MH, Chu SM, Hsu JF, Lien R, Huang HR, Chiang MC, et al Polymicrobial bloodstream infection in neonates: microbiology, clinical characteristics, and risk factors. PLoS One 2014; 9(1): p. e83082 10.1371/journal.pone.0083082 24454692 PMC3891628

[47] Chiesa C, Panero A, Osborn JF, Simonetti AF, Pacifico L. Diagnosis of neonatal sepsis: a clinical and laboratory challenge. Clin Chem. 2004; 50(2): p. 279–87. 10.1373/clinchem.2003.025171 14752012

